# *Burkholderia cepacia* Complex Regulation of Virulence Gene Expression: A Review

**DOI:** 10.3390/genes8010043

**Published:** 2017-01-19

**Authors:** Sílvia A. Sousa, Joana R. Feliciano, Tiago Pita, Soraia I. Guerreiro, Jorge H. Leitão

**Affiliations:** Institute for Bioengineering and Biosciences (IBB), and Department of Bioengineering, Instituto Superior Técnico, Universidade de Lisboa, Av. Rovisco Pais, 1049-001 Lisboa, Portugal; joana.feliciano@tecnico.ulisboa.pt (J.R.F.); t_pita@hotmail.com (T.P.); guerreiro.soraia25@gmail.com (S.I.G.)

**Keywords:** *Burkholderia cepacia* complex (Bcc), cystic fibrosis (CF), post-genomic approaches, adaptive strategies

## Abstract

*Burkholderia cepacia* complex (Bcc) bacteria emerged as opportunistic pathogens in cystic fibrosis and immunocompromised patients. Their eradication is very difficult due to the high level of intrinsic resistance to clinically relevant antibiotics. Bcc bacteria have large and complex genomes, composed of two to four replicons, with variable numbers of insertion sequences. The complexity of Bcc genomes confers a high genomic plasticity to these bacteria, allowing their adaptation and survival to diverse habitats, including the human host. In this work, we review results from recent studies using omics approaches to elucidate in vivo adaptive strategies and virulence gene regulation expression of Bcc bacteria when infecting the human host or subject to conditions mimicking the stressful environment of the cystic fibrosis lung.

## 1. Introduction

The *Burkholderia cepacia* complex (Bcc) is a group of 20 closely-related bacterial species, with up to 78% of their genes in common [1]. These bacteria have large genomes ranging from 7 to more than 9 Mbs, typically arranged in three chromosomes and a large plasmid [1,2]. Bcc emerged in the early 1980s as opportunistic human pathogens capable of causing particularly severe and life-threatening infections to cystic fibrosis (CF) patients [3,4]. Although all the Bcc species have the potential to infect CF patients, *Burkholderia cenocepacia* and *Burkholderia multivorans* account for the large majority of isolates from these patients [5,6]. Bcc infections are especially feared by CF patients due to the intrinsic resistance of these bacteria to current antimicrobial agents and the cepacia syndrome, a fast and often lethal necrotizing pneumonia accompanied with septicemia developed by up to 20% of the infected patients [7,8]. Epidemiology studies performed in the 1980s and 1990s showed that outbreaks in CF centers were mainly caused by particularly virulent and transmissible *B. cenocepacia* strains like the ET12, the PHDC, and the Midwest lineages [4]. Segregation measures adopted worldwide led to a reduction of *B. cenocepacia* prevalence. Most probably as a result of these measures, the emergence of non-clonal strains of Bcc species like *B. multivorans*, *Burkholderia contaminans*, and *Burkholderia dolosa* is now apparent.

Several virulence factors have been described for Bcc bacteria (reviewed in [4,5,9]). However, the exact role they play when the bacterium is infecting the CF host remains to be fully elucidated. In recent years, several studies have been performed aiming at the understanding of the strategies used by Bcc to survive and thrive in the CF host and how the bacterium adapts its gene expression program to survive the hostile environment that is the CF host. These studies make use of whole genome sequencing approaches and global transcriptomics analyses, only possible now due to the recent developments achieved in post-genomic techniques. Here we review selected studies based on the comparative genomics and/or quantitative transcriptomics of Bcc isolates when infecting the CF host.

## 2. Genomics and Transcriptomics Analysis in the Study of Bcc Pathogenesis

When infecting the airways of CF patients, Bcc bacteria experience stressful conditions, in particular those resulting from a challenging host immune defense, aggressive antimicrobial therapy, nutrient availability and oxygen limitation, among other stresses [10,11]. Despite the description of several virulence factors of the Bcc [4,5,12], the process of adaptation to the airways of CF patients is still poorly understood. Selected recent studies involving whole genome sequencing to characterize in vivo evolution of genomes, as well as transcriptomics analyses of Bcc when infecting the CF host or subject to conditions mimicking the CF lung conditions, are summarized in Table 1.

The airways of CF patients are prone to long-term bacterial infections by bacterial strains that persist for many years, allowing significant time for genetic adaptation [6]. During this process, bacterial pathogens can accumulate mutations that will allow them to adapt to the human host [20], evade the immune response [21], and become more resistant to antibiotic therapy [22]. Post-genomic methodologies such as comparative genomics analyses are powerful tools to identify genes that underwent selective pressure during infection in CF patients.

Using a post-genomic approach, Lieberman and colleagues (2011) identified genes under adaptive evolution by tracking frequent patterns of mutations in the same pathogenic *B. dolosa* strain during the infection of multiple CF patients [13]. Using this approach, 17 genes conserved in the *Burkholderia* genus were found as subject to positive selection. The genes accounting for the highest number of mutations were *gyrA*, the glycosyltransferase *wbaD*, and the *BDAG_01161* gene, and a homolog of *fixL*. These genes are involved in antibiotic resistance, O-antigen presentation and oxygen-dependent gene expression regulation, respectively [13]. Eleven of the mutated genes belong to functional categories already known to play a role in Bcc virulence, such as membrane synthesis, secretion, and antibiotic resistance [13]. However, the six remaining mutated genes were novel virulence determinants for Bcc and three of them (a glucoamylase, a sigma factor, and a methyltransferase) have no well-annotated homologs and have unclear roles in bacterial pathogenicity. The other three genes, homologs of *fnr*, *fixL*, and *fixJ*, are involved in oxygen-dependent gene expression regulation in bacteria and can be linked to the adaptation of the bacterium to the lowered oxygen tension in the CF mucus [23,24].

To understand if the beneficial mutations became fixed in the population or have an intermediate frequency and coexist with other lineages, Lieberman and colleagues studied the genotypic diversity of *B. dolosa* within CF patients by resequencing individual colonies and whole populations from single sputum samples [14]. These authors observed that, under strong selection, multiple adaptive mutations appeared, but none stay fixed in the population, originating diverse bacterial lineages that can coexist for many years.

*B. multivorans* is currently the predominant Bcc species infecting CF patients in France and Canada [6,25]. To identify the pathoadaptive mechanisms associated with long-term persistence of *B. multivorans* in the CF patient’s lungs, Silva and colleagues [15] sequenced the genomes of 22 *B. multivorans* isolates recovered over the 20 years of chronic colonization of a patient from whom isolates presented a mucoid-to-nonmucoid transition. Similarly to the evolutionary patterns observed for *B. dolosa*, results from this study also show that the adaptation of *B. multivorans* to the CF lung occurred through the slow accumulation of selected mutations. The genes identified as being under strong positive selection contained mutations that produced long-lived lineages (for instance, in the *rpfR* gene with the resulting elevated c-di-GMP intracellular levels), together with genes that accumulated mutations over time leading to altered key functions (e.g., *fixL*, *plsX*, *hfq*, *ompR*-like gene, *fis*-like gene). In addition, 10 *loci* bearing three or more independent mutations mainly involved in gene expression regulation and lipid metabolism were also identified. However, a frameshift in *mutL* that led to an increased mutation rate was observed for the last serial isolate, which represents an exception to the slow adaptation dynamics found for all the other isolates [15]. The emergence of hypermutators during evolution in CF lungs is a widespread phenomenon already reported for other bacterial pathogens, as well as for Bcc isolates from CF [26,27]. The accelerated evolutionary rates of mutator lineages have been previously shown to increase their antibiotic resistance, rendering their eradication more difficult [27].

A recent comparative transcriptomics and genomics study of two *B. contaminans* isolates recovered from blood and sputum revealed that the sputum isolate harbored mutations in *mutS* and *mutL*, indicating that adaptation to the patient lung led to a hypermutator phenotype [16].

To understand the adaptive responses that might occur during long-term infection with *B. cenocepacia* in the stressful environment of the CF lung under antimicrobial therapy, a comparative transcriptomics analysis was carried out for the initial *B. cenocepacia* isolate IST439 and its clonal variant IST4113, obtained three years later [17]. Using a 1.5-fold threshold as the cut-off value, 1024 genes were found as differentially expressed in these two variants, with 534 genes upregulated and 490 downregulated [17]. In this study, an important role for acyl-homoserine lactones (AHL)-mediated quorum sensing in the establishment of the chronic infection was noticed, with the *cciR* gene upregulated in the later isolate and 94 genes previously known as CciR-dependent exhibiting altered expression. For the later isolate, the upregulation of genes involved in translation, iron acquisition, drug efflux pumps, and cell envelope and outer membrane biogenesis were also observed. Upregulation of genes encoding amino acid transporters or catabolism, and downregulation of several cytochromes required for electron transfer under aerobic conditions, together with upregulation of genes required for microaerophilic conditions in the later isolate suggest an adaptation to the nutritional environment of the CF lung and to an oxygen-limited environment, respectively [17].

Bcc infections in CF patients usually cause chronic respiratory infections. Additionally, these infections pose a high risk for the development of the cepacia syndrome (CS) which is characterized by pulmonary exacerbation, high levels of inflammatory markers, and positive blood culture for Bcc [19]. Despite its clinical relevance, the mechanism underlying the onset and development of CS remains limited. In order to understand the bacterial factors associated with CS, the transcriptomic profile of isolates recovered from the blood and sputum of a patient with the CS was carried out [19]. The bloodstream bacterial isolates were found to express genes involved in the type III secretion system and the exopolysaccharide cepacian at higher levels and to suppress the activity of the flagellar system. It was also observed that changes in bacterial motility were almost exclusively observed in cases of *B. cenocepacia* infection that resulted in CS. The loss of motility was observed in isolates recovered from the sputum up to 24 months before the development of CS [19].

Aiming at the study of the natural evolution of the ET12 epidemic lineage circulating in the CF community since the recovery of the J2315 strain in 1989 and mapping the multiple antimicrobial resistance pathways of J2315, the transcriptomics analysis of two pan-resistant ET12 outbreak isolates recovered two decades after J2315 were compared with the transcriptome of J2315 [18]. Genes involved in flagella production and in chemotaxis were downregulated in the outbreak isolates. Genes involved in transport and efflux, restriction modification, and transposition were upregulated in the outbreak isolates. In particular, the gene *BCAS0081* encoding an ABC membrane transport protein probably involved in antimicrobial resistance was highly expressed [18].

Several transcriptomics analyses of Bcc strains provided new knowledge on non-coding regulatory RNAs (sRNAs) [28], differential gene expression in CF sputum [29], and in artificial sputum versus soil-based medium [30,31], and in nine different growth conditions mimicking conditions that can occur in the environment or in the CF lung [11]. These studies have also contributed to the identification of several genes involved in quorum sensing regulation [32,33,34], antibiotic resistance [18,35], and biofilm formation and resistance [36,37,38,39].

## 3. Known Regulators of Bcc Virulence Factors

In spite of the huge amount of data generated from comparative genomics and transcriptomics studies, the exact function of specific genes is often limited. In addition, about 15% of the genes of *B. cenocepacia* J2315 (one of the best studied Bcc strains) still have no predicted function or only a general function predicted. Bcc bacteria occupy a diverse array of niches, including the rhizosphere, plants, animals and humans [5], requiring finely-tuned regulatory mechanisms that enable them to adapt to diverse environments and different stresses. In this section, we present a summarized description of known regulators of virulence of Bcc bacteria.

### 3.1. Sigma Factors and Related Proteins

During bacterial evolution, selective pressure caused by different environmental conditions has been shown to favor the emergence of several paralogous lineages of σ^70^ [40]. In the ET12 lineage strain *B. cenocepacia* J2315, 20 σ^70^ paralog genes were identified, with 14 of them belonging to the extracytoplasmic function (ECF) family [40]. These identified σ^70^ paralogs were shown to have a clade-specific distribution, with *ecfA2* and *sigI* restricted to ET12 lineage strains and Bcc species, respectively [40]. Some σ^70^ paralogous genes are only present in the *Burkholderia* genus (*ecfJ*, *ecfF* and *sigJ*), or in proteobacteria (*ecfA1*, *ecfC*, *ecfD*, *ecfE*, *ecfG*, *ecfL*, *ecfM*, and *rpoS*). Common eubacterial σ^70^ paralogous genes (*ecfI*, *ecfK*, *ecfH*, *ecfB*, and *rpoD*-, *rpoH*-, *fliA*-like genes) were also identified.

It was shown by transcriptomic assays that four sigma factors (BCAL0787 or σ^32^, BCAL1369 or FecI, BCAL1688 or OrbS, and BCAL3478) were upregulated in sessile cells of *B. cenocepacia* J2315 in response to oxidative stress [36].

A mechanism used by bacteria to adapt to stress involves the activity of the alternative sigma factor RpoE, an important regulator of the extracytoplasmic stress response [41]. A *B. cenocepacia* K56-2 *rpoE* mutant was found to present cell envelope alterations, exhibiting growth defects under elevated temperature and osmolarity [42]. RpoE was also shown to be required for the *B. cenocepacia* ability to delay phagolysosomal fusion in macrophages, allowing the bacteria to survive intracellularly [42]. Results from the mapping of the σ^54^ regulon and the characterization of a σ^54^ mutant derived from *B. cenocepacia* H111 suggest that this alternative sigma factor plays an important role in the control of nitrogen metabolism, in the metabolic adaptation to stressful and nutrient-limited environments, and also in the virulence of this strain towards the *Caenorhabditis elegans* infection model [43]. In addition to genes involved in nitrogen metabolism, other genes were activated by low nitrogen in a σ^54^-dependent manner, namely the two gene clusters involved in the biosynthesis of the exopolysaccharide Cepacian. Cepacian was previously shown to protect cells from external stresses, such as desiccation and metal ion stress [44], and also to be involved in pathogenic interactions [45,46], to interfere with the innate host immune system [45,47], being required for the formation of thick and mature biofilms [48]. Additionally, RpoN was also found to regulate genes involved in biofilm formation, motility, and virulence [43,49]. In *B. cenocepacia* K56-2, RpoN was shown to be important for the intracellular trafficking and survival of the bacteria within infected macrophages [49]. Unlike other alternative sigma factors, RpoN-dependent gene transcription initiation requires an enhancer binding protein that is often a response regulator belonging to a two-component regulatory system [49]. Despite the existence of 20 predicted enhancer binding proteins in *B. cenocepacia*, a mutant defect in one of these proteins (BCAL1536) exhibited an attenuated virulence in a rat agar bead infection model [50].

### 3.2. Quorum Sensing

Quorum sensing (QS) is a form of bacterial cell-to-cell communication that is mediated by the production of signaling molecules, called autoinducers [51]. The autoinducers accumulate outside the cell and once their concentration reaches a critical value (the threshold), specific receptors mediate the induction or repression of target gene expression [51]. The LuxIR homolog CepIR was the first QS system to be identified in a *B. cenocepacia* strain [52]. Since then, the CepIR was shown to be widespread in Bcc bacteria [53,54], being required for full virulence in Bcc infection models including *C. elegans*, *Galleria mellonella*, rodents, zebrafish, alfalfa, and onions [55,56,57]. The CepIR system relies on the acyl homoserine lactone (AHL) synthase CepI and the transcriptional regulator CepR that specifically binds to AHL becoming active. In its activated form, CepR binds to the cep boxes, located upstream specific genes, causing the induction or repression of their expression [51,54]. CepI synthesizes N-octanoylhomoserine lactone (C_8_-HSL) and, as a minor by-product, *N*-hexanoylhomoserine lactone (C_6_-HSL) as signalling molecules [51,54]. The CepIR system has been shown to be involved in the regulation of toxins, lipases, extracellular proteases, iron-chelating siderophores, antifungal agents, swarming motility, and biofilm formation (reviewed in [51,57,58,59]). In *Burkholderia vietnamiensis*, a *N*-decanoyl homoserine lactone (C10-HSL)-dependent QS system named BviIR, was also described [60].

A QS system based on the fatty acid molecule *cis*-2-dodecenoic acid as the signaling molecule was identified for the first time in *B. cenocepacia* J2315 and was named *Burkholderia* diffusible signal factor (BDSF) [61]. This molecule is used as the signaling molecule of the RpfFR QS system highly conserved within Bcc [62]. The RpfFR system relies on the biosynthesis of BDSF by the bifunctional crotonase RpfF and the BDSF receptor protein RpfR containing PAS-GGDEF-EAL domains [59]. The signaling molecule BDSF binds to RpfR, stimulating the cyclic dimeric guanosine monophosphate (c-di-GMP) phosphodiesterase activity of the protein, and thus lowering the intracellular c-di-GMP levels [63]. This QS system interferes with cell motility, biofilm formation, proteolytic activity, and virulence [59,63]. Several of the genes positively regulated by RpfFR are also controlled by the CepIR QS system [34]. However, the contribution of each of the QS systems to the regulation of target genes was found to be variable and their functions seem to be parallel [34]. Molecules of the DSF-family, including BDSF, are involved in interspecies and interkingdom communication [61,64]. Recently, molecules of this family were found in the sputum of CF patients, suggesting that bacterial interspecies communication mediated by DSF molecules can occur in the CF lung [65].

*B. cenocepacia* in particular has two additional QS systems: the AHL-type system CciIR (encoded on a genetic *locus* known as the *B. cenocepacia* island (*cci*) and found in ET12 lineage strains) [66] and the CepR2 [57]. The AHL synthase CciI synthesizes C_6_-HSL and, in minor amounts, C_8_-HSL that can be bound to the receptor CciR [66]. The CciIR and CepIR systems can interact with each other, with CciR negatively regulating *cepI* and CepR regulating positively *cciI* [66]. CepR2, an orphan LuxR homolog, was shown to act as an anti-activator of CepS, an AraC-type regulator, that activates gene expression in the absence of CepR2 [67].

Additional regulators affecting the QS circuitry in Bcc strains have been identified. For instance, the LysR-type transcriptional regulator ShvR (**S**hiny colony variants Regulator) was shown to negatively control *cepIR* and *cciIR* expression while positively affecting biofilm formation [68]. The membrane hybrid sensor kinase AtsR (Adhesion and type six secretion system Regulator) was shown to be a negative regulator of *cepI* and *cciI* expression [69]. Recently, AtsR was also shown to be a global regulator, controlling the expression of virulence factors in the absence of the CepIR QS system [70]. The upregulation of *cepI* in response to low oxygen concentration was found as independent of cell density [11]. However, the mechanism responsible for QS regulation under low concentration of oxygen remains unknown.

### 3.3. Iron Acquisition and Bcc Virulence

Iron is an essential element for living cells, it is present in cytochromes, ferredoxins, and other iron-containing proteins, and it is also a co-factor of several enzymes. When infecting mammalian hosts, bacterial pathogens face a shortage of iron, since this metal ion is bound to transferrin and lactoferrin. To fulfill their iron requirements, Bcc bacteria use metal chelator molecules named siderophores to capture and transport iron into the bacterial cell, using specialized receptors localized in the membrane. Therefore, although iron acquisition is not a virulence factor per se, systems used to acquire iron in the context of interaction with the host ultimately play a role in virulence. Several iron acquisition mechanisms by Bcc are known [71]. Bcc bacteria can produce up to four siderophores (ornibactin, pyochelin, cepabactin, and cepaciachelin), although evidence points out ornibactins as the most important [72]. A mutant in *orbA*, one of the three genes involved in ornibactin biosynthesis, was shown to be impaired in virulence in an animal model [73]. Genes for ornibactin synthesis and utilization were found as significantly upregulated when cells were exposed to iron limited availability [74]. Furthermore, data was also presented by these authors, indicating that iron acquisition during chronic lung infection is multifaceted, and *B. cenocepacia* has the ability to differentially activate the expression of genes for iron acquisition in the presence of the major CF pathogen and iron competitor *Pseudomonas aeruginosa*.

Bcc bacteria are also endowed with iron acquisition mechanisms independent of siderophore production [71]. Whitby et al. [75] showed that *B. cenocepacia* J2315 uses ferritin in a protease-dependent manner. Several *B. cenocepacia* strains are also able to use hemin as an iron source [74,76]. Heme is a component of hemoglobin and the most abundant iron source in the host body. Therefore, Bcc strains that produce hemolysins can have access to this source of iron. Using ram erythrocytes, Bevivino et al., (2002) [77] found that the majority of the *Burkholderia ambifaria* isolates tested were hemolytic. However, the percentage of hemolytic *B. cenocepacia* isolates from environmental sources was higher than those of clinical origin. Hutchison et al. (1998) identified a lipopeptide secreted by *B. cenocepacia* capable of erythrocyte hemolysis [78]. Thomson and Dennis (2012) [79] identified a gene cluster involved in the expression of a toxin with β-hemolytic activity required for full virulence of Bcc in the *Galeria mellonella* model of infection. The toxin was shown to be synthesized via a non-ribosomal peptide synthetase (NRPS) mechanism. However, in this study, production of NRPS-derived occidiofungin/burkholdine-like compounds was shown to be limited to *B. ambifaria*, *B. contaminans*, *B. pyrrocinia*, and *B. vietnamiensis.* The fact that this toxin was not found in the most common clinical Bcc species, *B. cenocepacia* and *B. multivorans*, suggested to the authors that this gene cluster evolved to protect Bcc bacteria from ecological niche predators such as amoeba and fungi [79].

### 3.4. Activators and Repressors

Adaptive responses are commonly mediated by transcriptional regulators. In most of the cases, the regulators involved in transcriptional control are two-domain proteins with a signal receiving domain and a DNA-binding domain which transduces the signal [80]. The bioinformatics analysis of Bcc genome sequences revealed several genes putatively codifying for various families of transcriptional regulators, including the AraC, ArsR, AsnC, DeoR, GntR, IcIR, LacI, LysR, MarR, MerR, and TetR families [81]. However, only a few were already functionally characterized.

For instance, the LysR-type transcriptional regulator, ShvR, was shown to regulate over 1000 genes, affecting Bcc colony morphology, biofilm formation, virulence in plant and animal infection models, and some quorum-sensing-dependent phenotypes [68,82,83]. In particular, ShvR downregulates the expression of *cepIR* and *cccIR*, type II secretion system, proteases, and lipases [82]. In addition, ShvR is an inducer of biofilm formation and of rough colony morphology in a CepR-independent mechanism [82]. ShvR was also shown to directly regulate the expression of the promoters of *afcA* and *afcC* genes required for the production of an antifungal agent [82]. Subramoni and colleagues [84] have shown that ShvR also regulates *afcE* and *afcF* encoding an acyl-CoA dehydrogenase and a Flavin adenine dinucleotide (FAD)-dependent oxidoreductase, respectively. In their work, these authors have also shown that these two proteins are required for the synthesis of a membrane-associated antifungal lipopeptide, affecting the membrane morphology and permeability, the surface motility, and the total cellular lipid composition. The *shvR* expression is positively-regulated by CepIR and CciIR in a feedback loop of regulation [32].Recently, a novel transcriptional regulator, named BcRsaM was identified in Bcc [85]. BcRsaM was postulated to be involved in the modulation of CepI abundance and CepR activity [85].

### 3.5. Two-Component Regulatory Systems

Two-component regulatory systems (TCSs) are composed by two proteins, a membrane-bound histidine kinase that receives the environmental stimuli and autophosphorylates and then transfers the phosphate to a cytoplasmic DNA-binding response regulator that mediates changes in gene expression [86].

The bioinformatics analysis of Bcc genome sequences revealed at least 28 putative two-component systems, however only a few are already functionally characterized [81]. Recently, the *B. cenocepacia* K56-2 BceSR regulatory system was described [87]. A *bceR* mutant exhibited a decreased protease production, quorum sensing, and swimming motility, and reduced virulence in the alfalfa plant model [87]. Bioinformatics analysis of other Bcc genome sequences revealed that the BceSR system is also present in *B. ambifaria*, *B. multivorans*, *B. vietnamiensis*, and *B. dolosa* [87]. A novel TCS was recently identified in *B. cenocepacia* and was named *esaSR* [88]. The deletion of the response regulator *esaR* caused the sensibility of *B. cenocepacia* to carbonyl cyanide 3-chlorophenylhydrazone, chloramphenicol, ciprofloxacin, kanamycin, meropenem, novobiocin, and tetracycline [88]. This mutation also led to a defect in the membrane integrity [88].

The AtsR/AtsT phosphorelay pathway was shown to be a major global regulator of *B. cenocepacia* pathogenicity [70]. This mechanism was shown to negatively regulate quorum sensing, biofilm production, type VI secretion system (T6SS), and protease secretion [70]. Deletion of *atsR* was shown to cause a significant increase in T6SS activity that induces actin cytoskeletal rearrangements of infected macrophages and delay the assembly of the NADPH oxidase complex at the membrane of the *B. cenocepacia*-containing vacuole [89]. Flanagan and colleagues [90] identified a six-gene cluster encoding a TCS (*BCAL2831* and *BCAL2830*) and an HtrA protease (*BCAL2829*). Mutation of the *BCAL2831* affected the gene cluster transcription, revealing that the encoding proteins are required for osmotic and prolonged heat stress and for survival in a rat model of chronic lung infection [90]. The lack of complementation of the *BCAL2831* mutation by expression of the High temperature requirement A (HtrA) protease revealed that the TCS identified could regulate the genes required for growth under stress conditions and for survival in vivo [90].Recently, the oxygen-sensing two-component regulatory system FixLJ was identified in *B. dolosa* as being involved in the regulation of motility, biofilm formation, intracellular invasion of macrophages, and virulence [91].

### 3.6. Toxin-Antitoxin

A total of 16 toxin-antitoxin (TA) systems were recently identified in the *B. cenocepacia* J2315 genome, with only 9 of them common to other Bcc strains [37]. Bacterial TA loci consist of two genes, one encoding a potentially toxic protein, and the other encoding an antitoxin that represses the toxin function or expression [92]. The antitoxin can be either a protein or a RNA. TA systems from other bacterial species have been described as regulators of biofilm formation, programmed cell death or inhibition of cell growth, stabilization of mobile genetic elements, and persistence [93]. The upregulation of toxin-antitoxin genes in the adaptation process to growth arrest caused by both stationary phase and low-oxygen conditions was reported based on transcriptomics methodologies [11].

In *B. cenocepacia*, the overexpression of seven of these toxins (BCAL0610, BCAM0272, BCAM0627, BCAS0580, pBCA093, BCAL0070, and BCAL3209) led to the development of persister cells after exposure to ciprofloxacin or tobramycin [37]. A correlation between the growth delay and the development of persister cells was observed. The overexpression of some toxins also led to alterations in biofilm formation [37]. Therefore, the expression of TA systems was compared in antibiotic-treated and untreated planktonic and sessile cultures. This comparison showed that the expression of nine toxin-encoding genes was higher after treatment with tobramycin. However, no changes occurred after treatment with ciprofloxacin, revealing that specific TA modules contribute to *B. cenocepacia* persistence, as their upregulation is specifically dependent on the mode of growth of the bacterium and of the antibiotic used [37].

### 3.7. sRNA and Hfq

When comparing the transcriptome of two related strains of *B. cenocepacia* (of clinical and environmental origins) under conditions that mimic CF sputum versus soil, Yoder-Himes et al. [31] noticed that a high number of transcripts induced in *B. cenocepacia* J2315 originated from intergenic regions. This observation indicates that the identified transcripts are either non-coding RNAs (sRNAs) or unannotated genes that encode for mRNAs. sRNAs are bacterial key posttranscriptional regulators of gene expression affecting a wide range of cellular processes, including virulence in several bacterial pathogens (reviewed in [94,95]). A large class of sRNAs exert their regulatory action by base pairing with specific mRNAs (the mRNA targets) with the help of the RNA chaperone Hfq. The sRNA-mRNA double stranded complex often hinders the ribosome binding site (RBS) of the mRNA, leading to repression of gene expression [96]. In a few cases, the double-stranded complex exposes the RBS of the mRNA, resulting in increased expression of the encoded protein [96].

In a bioinformatics study, Coenye et al. [28] identified 213 putative sRNAs on the genome of the highly epidemic clinical isolate *B. cenocepacia* J2315, although the authors have only confirmed the expression of four of these sRNAs. The comparison of the transcriptional responses of *B. cenocepacia* J2315 growing as planktonic or sessile upon exposure to chlorhexidine also revealed that 19 intergenic (IG) sequences putatively encoding sRNAs were differentially expressed, suggesting a putative role of these sRNA on chlorhexidine tolerance [39]. However, their biological function remains unknown. Several IG were also shown to be differentially expressed by sessile cells of *B. cenocepacia* J2315 subject to oxidative stress [36].

The Hfq protein is an RNA chaperone that mediates interactions between sRNAs and their target mRNAs, playing an important role as a global regulator of bacterial metabolism and virulence (recently reviewed in [96,97]). Together with a few other prokaryotes, Bcc bacteria encode in their genomes two distinct and functional Hfq-like proteins, *hfq* and *hfq2* genes [98]. A co-purification experiment of the RNA binding Hfq protein and total RNA from *B. cenocepacia* J2315 led to the identification and validation of 24 novel sRNAs, not previously identified by transcriptomics or bioinformatics studies [99]. The *cis*-encoded h2cR and the *trans*-encoded MtvR sRNAs remain as the few Bcc sRNAs functionally characterized [100,101]. The h2cR sRNA was shown to negatively regulate *hfq2* mRNA by binding to the 5′-UTR region of the *hfq2* mRNA, leading to an accelerated decay of *hfq2* mRNA and reduced protein levels in exponentially growing cells [100]. MtvR was described as conserved in Bcc, acting as a global regulator affecting bacterial growth, motility, biofilm formation, virulence, and resistance to stress and antibiotics [101].

A recent study based on a genome-wide mapping of transcription start sites of biofilm-grown *B. cenocepacia* J2315 revealed that 15 non-coding sRNAs were highly expressed under those conditions [38]. Homologs to 9 out of these 15 putative non-coding sRNAs were found in the RFAM database. Interestingly, four of these sRNA belong to the new ‘toxic sRNAs’ family, known due to their toxicity when overexpressed in *E. coli.* Their presence in the genomes of four strains of *B. cenocepacia* was confirmed by Northern-blot [102]. Expression of the other six non-coding sRNAs was previously experimentally confirmed [30,99], but their function, as for most of the Bcc sRNAs, is still unknown.

## 4. Conclusions

**Figure 1:**
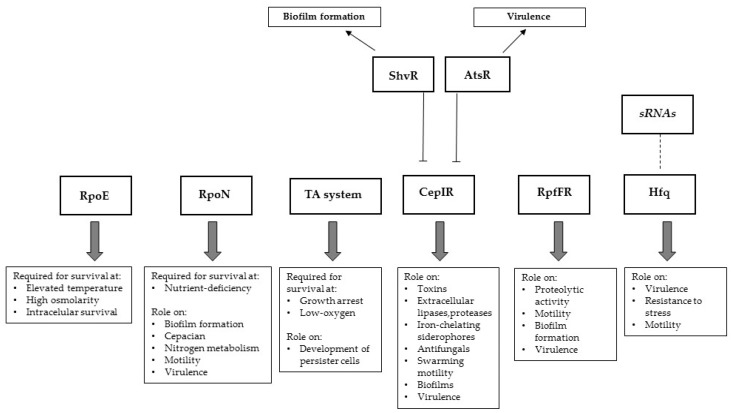
Schematic representation of *Burkholderia cepacia* complex (Bcc) regulators that emerged as involved in virulence and other phenotypes based on transcriptomics studies using bacteria infecting cystic fibrosis (CF) patients or cultivated under conditions mimicking the CF lung environment. Despite the description of several sRNAs actively involved in Bcc virulence, their specific function remains to be fully characterized and therefore a dashed-line linking sRNAs and Hfq was used.

The recent use of high-throughput sequencing and “omics” strategies sheds new light on the mechanisms used by Bcc bacteria to adapt to the CF lung. From the still-limited comparative genomics studies performed using Bcc serial isolates, the emerging picture is that adaptive mutations lead to the co-evolution of multiple lineages in the CF lung. Genes identified as subject to a strong in vivo selection include those involved in antibiotic resistance, adaptation to low oxygen, iron acquisition, adaptation to the biofilm lifestyle, and adherence to the respiratory epithelial surface. Transcriptomics studies also point out differentially expressed genes involved in adaptation to low oxygen availability, transport and efflux systems, quorum sensing, exopolysaccharide, and flagella biosynthesis, as a result of lung adaptation. These mechanisms include the sensing of alterations in the environment during the infection process and the use of a combination of regulatory systems to survive in the host (Figure 1). This complex pattern of regulatory interplay between diverse regulatory mechanisms is only emerging and new data expected to contribute to our understanding of Bcc infections will certainly be revealed in the near future.

## Figures and Tables

**Table 1:** Summary of major findings obtained in selected genomics and transcriptomics studies related to *Burkholderia cepacia* complex pathogenesis in the cystic fibrosis (CF) lung.

Experiment	Major Findings	Reference
**Genomics**		
Retrospective study of a *B. dolosa* outbreak among CF patients by sequencing the genomes of a single colony of 112 isolates collected from 14 individuals over 16 years.	17 bacterial genes acquired non-synonymous mutations in multiple individuals, indicating parallel adaptive evolution. Mutated genes involved in antibiotic resistance, bacterial membrane composition, and oxygen-dependent gene expression regulation.	Lieberman et al., 2011 [13]
Analysis of intraspecies diversity in the sputum of five CF patients infected with *B. dolosa*.	Several bacterial lineages coexisted within the same patient. Genes subject to strongest selection are involved in outer membrane component synthesis, iron scavenging and antibiotic resistance.	Lieberman et al., 2014 [14]
Genomic and functional evolution analysis of 22 *B. multivorans* isolates sequentially collected over 20 years.	Population diversified into four primary, coexisting clades, with distinct evolutionary dynamics. Strong selection of genes involved in *B. multivorans* populations during long-term colonization of CF patient lung targets adherence, metabolism, and cell envelope changes related to adaptation to the biofilm lifestyle.	Silva et al., 2016 [15]
Genome-wide comparative analysis of two *B. contaminans* isolates, from sputum and from blood culture.	The sputum isolate differs from the bloodstream isolate by over 1400 mutations as a result of a mismatch repair-deficient hypermutable state.	Nunvar et al., 2016 [16]
**Transcriptomics**		
Comparative transcriptomics of two clonal isolates of *B. cenocepacia* in different stages of chronic infection, recovered from a long-term colonized CF patient deceased with cepacia syndrome.	The later isolate presented upregulated genes involved in translation, iron acquisition, efflux of drugs, and adhesion to respiratory epithelial surface. Alterations related to adaptation to the nutritional environment of the CF lung and to oxygen-limitation.	Mira et al., 2011 [17]
Comparative transcriptomics of two pan-resistant ET12 outbreak isolates recovered two decades after J2315.	The outbreak strains exhibited downregulation of genes involved in flagella production and chemotaxis; upregulation of genes involved in transport and efflux, restriction modification, and transposition.	Sass et al., 2011 [18]
Comparative transcriptomics of *B. cenocepacia* isolates from the bloodstream of CF patients with cepacia syndrome with isolates recovered from sputum one to two months before the cepacia syndrome.	Blood isolates presented a higher level of expression of virulence genes involved in type III secretion, exopolysaccharide cepacian biosynthesis, and quorum sensing, and reduced expression of flagellar genes.	Kalferstova et al., 2015 [19]
Comparative transcriptomics of two *B. contaminans* isolates from sputum and blood culture of a CF patient.	Differential expression of quorum sensing-regulated virulence factors, motility, and chemotaxis; the low-oxygen-activated (*lxa*) locus encoding stress-related proteins; and two clusters responsible for the biosynthesis of the antifungal and hemolytic compounds pyrrolnitrin and occidiofungin.	Nunvar et al., 2016 [16]

## References

[1] Holden M.T., Seth-Smith H.M., Crossman L.C., Sebaihia M., Bentley S.D., Cerdeno-Tarraga A.M., Thomson N.R., Bason N., Quail M.A., Sharp S. (2009). The Genome of *Burkholderia cenocepacia* J2315, an Epidemic Pathogen of Cystic Fibrosis Patients. J. Bacteriol..

[2] Ussery D.W., Kiil K., Lagesen K., Sicheritz-Pont-eacuten T., Bohlin J., Wassenaar T.M. (2009). The Genus *Burkholderia*: Analysis of 56 Genomic Sequences. Microbial Pathogenomics.

[3] Depoorter E., Bull M.J., Peeters C., Coenye T., Vandamme P., Mahenthiralingam E. (2016). *Burkholderia*: An update on taxonomy and biotechnological potential as antibiotic producers. Appl. Microbiol. Biotechnol..

[4] Mahenthiralingam E., Urban T.A., Goldberg J.B. (2005). The multifarious, multireplicon *Burkholderia cepacia* complex. Nat. Rev. Microbiol..

[5] Leitão J.H., Sousa S.A., Ferreira A.S., Ramos C.G., Silva I.N., Moreira L.M. (2010). Pathogenicity, virulence factors, and strategies to fight against *Burkholderia cepacia* complex pathogens and related species. Appl. Microbiol. Biotechnol..

[6] Lipuma J.J. (2010). The changing microbial epidemiology in cystic fibrosis. Clin. Microbiol. Rev..

[7] Leitão J.H., Sousa S.A., Cunha M.V., Salgado M.J., Melo-Cristino J., Barreto M.C., Sá-Correia I. (2008). Variation of the antimicrobial susceptibility profiles of *Burkholderia cepacia* complex clonal isolates obtained from chronically infected cystic fibrosis patients: A five-year survey in the major Portuguese treatment center. Eur. J. Clin. Microbiol. Infect. Dis..

[8] Jones A.M., Dodd M.E., Webb A.K. (2001). *Burkholderia cepacia*: Current clinical issues, environmental controversies and ethical dilemmas. Eur. Respir. J..

[9] Loutet S.A., Valvano M.A. (2010). A decade of *Burkholderia cenocepacia* virulence determinant research. Infect. Immun..

[10] Yang L., Jelsbak L., Molin S. (2011). Microbial ecology and adaptation in cystic fibrosis airways. Environ. Microbiol..

[11] Sass A.M., Schmerk C., Agnoli K., Norville P.J., Eberl L., Valvano M.A., Mahenthiralingam E. (2013). The unexpected discovery of a novel low-oxygen-activated locus for the anoxic persistence of *Burkholderia cenocepacia*. ISME J..

[12] Saldías M.S., Valvano M.A. (2009). Interactions of *Burkholderia cenocepacia* and other *Burkholderia cepacia* complex bacteria with epithelial and phagocytic cells. Microbiology.

[13] Lieberman T.D., Michel J.-B., Aingaran M., Potter-Bynoe G., Roux D., Davis M.R., Skurnik D., Leiby N., LiPuma J.J., Goldberg J.B. (2011). Parallel bacterial evolution within multiple patients identifies candidate pathogenicity genes. Nat. Genet..

[14] Lieberman T.D., Flett K.B., Yelin I., Martin T.R., McAdam A.J., Priebe G.P., Kishony R. (2014). Genetic variation of a bacterial pathogen within individuals with cystic fibrosis provides a record of selective pressures. Nat. Genet..

[15] Silva I.N., Santos P.M., Santos M.R., Zlosnik J.E.A., Speert D.P., Buskirk S.W., Bruger E.L., Waters C.M., Cooper V.S., Moreira L.M. (2016). Long-Term Evolution of *Burkholderia multivorans* during a Chronic Cystic Fibrosis Infection Reveals Shifting Forces of Selection. mSystems.

[16] Nunvar J., Kalferstova L., Bloodworth R.A.M., Kolar M., Degrossi J., Lubovich S., Cardona S.T., Drevinek P., Bevivino A. (2016). Understanding the Pathogenicity of *Burkholderia contaminans*, an Emerging Pathogen in Cystic Fibrosis. PLoS ONE.

[17] Mira N.P., Madeira A., Moreira A.S., Coutinho C.P., Sá-Correia I. (2011). Genomic Expression Analysis Reveals Strategies of *Burkholderia cenocepacia* to Adapt to Cystic Fibrosis Patients’ Airways and Antimicrobial Therapy. PLoS ONE.

[18] Sass A., Marchbank A., Tullis E., Lipuma J.J., Mahenthiralingam E. (2011). Spontaneous and evolutionary changes in the antibiotic resistance of *Burkholderia cenocepacia* observed by global gene expression analysis. BMC Genom..

[19] Kalferstova L., Kolar M., Fila L., Vavrova J., Drevinek P. (2015). Gene expression profiling of *Burkholderia cenocepacia* at the time of cepacia syndrome: Loss of motility as a marker of poor prognosis?. J. Clin. Microbiol..

[20] Smith E.E., Buckley D.G., Wu Z., Saenphimmachak C., Hoffman L.R., D’Argenio D.A., Miller S.I., Ramsey B.W., Speert D.P., Moskowitz S.M. (2006). Genetic adaptation by *Pseudomonas aeruginosa* to the airways of cystic fibrosis patients. Proc. Natl. Acad. Sci. USA.

[21] Deitsch K.W., Lukehart S.A., Stringer J.R. (2009). Common strategies for antigenic variation by bacterial, fungal and protozoan pathogens. Nat. Rev. Microbiol..

[22] Rau M.H., Hansen S.K., Johansen H.K., Thomsen L.E., Workman C.T., Nielsen K.F., Jelsbak L., Høiby N., Yang L., Molin S. (2010). Early adaptive developments of *Pseudomonas aeruginosa* after the transition from life in the environment to persistent colonization in the airways of human cystic fibrosis hosts. Environ. Microbiol..

[23] Crosson S., McGrath P.T., Stephens C., McAdams H.H., Shapiro L. (2005). Conserved modular design of an oxygen sensory/signaling network with species-specific output. Proc. Natl. Acad. Sci. USA.

[24] Worlitzsch D., Tarran R., Ulrich M., Schwab U., Cekici A., Meyer K.C., Birrer P., Bellon G., Berger J., Weiss T. (2002). Effects of reduced mucus oxygen concentration in airway *Pseudomonas* infections of cystic fibrosis patients. J. Clin. Investig..

[25] Govan J.R., Brown A.R., Jones A.M. (2007). Evolving epidemiology of *Pseudomonas aeruginosa* and the *Burkholderia cepacia* complex in cystic fibrosis lung infection. Future Microbiol..

[26] Martina P., Feliziani S., Juan C., Bettiol M., Gatti B., Yantorno O., Smania A.M., Oliver A., Bosch A. (2014). Hypermutation in *Burkholderia cepacia* complex is mediated by DNA mismatch repair inactivation and is highly prevalent in cystic fibrosis chronic respiratory infection. Int. J. Med. Microbiol..

[27] Oliver A., Mena A. (2010). Bacterial hypermutation in cystic fibrosis, not only for antibiotic resistance. Clin. Microbiol. Infect..

[28] Coenye T., Drevinek P., Mahenthiralingam E., Shah S.A., Gill R.T., Vandamme P., Ussery D.W. (2007). Identification of putative noncoding RNA genes in the *Burkholderia cenocepacia* J2315 genome. FEMS Microbiol. Lett..

[29] Drevinek P., Holden M.T.G., Ge Z., Jones A.M., Ketchell I., Gill R.T., Mahenthiralingam E. (2008). Gene expression changes linked to antimicrobial resistance, oxidative stress, iron depletion and retained motility are observed when *Burkholderia cenocepacia* grows in cystic fibrosis sputum. BMC Infect. Dis..

[30] Yoder-Himes D.R., Chain P.S.G., Zhu Y., Wurtzel O., Rubin E.M., Tiedje J.M., Sorek R. (2009). Mapping the *Burkholderia cenocepacia* niche response via high-throughput sequencing. Proc. Natl. Acad. Sci. USA.

[31] Yoder-Himes D.R., Konstantinidis K.T., Tiedje J.M. (2010). Identification of potential therapeutic targets for *Burkholderia cenocepacia* by comparative transcriptomics. PLoS ONE.

[32] O’Grady E.P., Viteri D.F., Malott R.J., Sokol P.A. (2009). Reciprocal regulation by the CepIR and CciIR quorum sensing systems in *Burkholderia cenocepacia*. BMC Genom..

[33] Inhülsen S., Aguilar C., Schmid N., Suppiger A., Riedel K., Eberl L. (2012). Identification of functions linking quorum sensing with biofilm formation in *Burkholderia cenocepacia* H111. Microbiologyopen.

[34] Schmid N., Pessi G., Deng Y., Aguilar C., Carlier A.L., Grunau A., Omasits U., Zhang L.H., Ahrens C.H., Eberl L. (2012). The AHL- and BDSF-Dependent Quorum Sensing Systems Control Specific and Overlapping Sets of Genes in *Burkholderia cenocepacia* H111. PLoS ONE.

[35] Bazzini S., Udine C., Sass A., Pasca M.R., Longo F., Emiliani G., Fondi M., Perrin E., Decorosi F., Viti C. (2011). Deciphering the role of rnd efflux transporters in *Burkholderia cenocepacia*. PLoS ONE.

[36] Peeters E., Sass A., Mahenthiralingam E., Nelis H., Coenye T. (2010). Transcriptional response of *Burkholderia cenocepacia* J2315 sessile cells to treatments with high doses of hydrogen peroxide and sodium hypochlorite. BMC Genom..

[37] Van Acker H., Sass A., Dhondt I., Nelis H.J., Coenye T. (2014). Involvement of toxin-antitoxin modules in *Burkholderia cenocepacia* biofilm persistence. Pathog. Dis..

[38] Sass A.M., van Acker H., Förstner K.U., van Nieuwerburgh F., Deforce D., Vogel J., Coenye T. (2015). Genome-wide transcription start site profiling in biofilm-grown *Burkholderia cenocepacia* J2315. BMC Genom..

[39] Coenye T., van Acker H., Peeters E., Sass A., Buroni S., Riccardi G., Mahenthiralingam E. (2011). Molecular mechanisms of chlorhexidine tolerance in *Burkholderia cenocepacia* biofilms. Antimicrob. Agents Chemother..

[40] Menard A., de los Santos P.E., Arnault Graindorge V., Cournoyer B., Genomics B., Cournoyer A. (2007). Architecture of *Burkholderia cepacia* complex sigma 70 gene family: Evidence of alternative primary and clade-specific factors, and genomic instability Architecture 70 alternative. BMC Genom..

[41] Raina S., Missiakas D., Georgopoulos C. (1995). The *rpoE* gene encoding the sigma E (sigma 24) heat shock sigma factor of *Escherichia coli*. EMBO J..

[42] Flannagan R.S., Valvano M.A. (2008). *Burkholderia cenocepacia* requires RpoE for growth under stress conditions and delay of phagolysosomal fusion in macrophages. Microbiology.

[43] Lardi M., Aguilar C., Pedrioli A., Omasits U., Suppiger A., Cárcamo-Oyarce G., Schmid N., Ahrens C.H., Eberl L., Pessi G. (2015). σ54-Dependent Response to Nitrogen Limitation and Virulence in *Burkholderia cenocepacia* Strain H111. Appl. Environ. Microbiol..

[44] Ferreira A.S., Leitão J.H., Silva I.N., Pinheiros P.F., Sousa S.A., Ramos C.G., Moreira L.M. (2010). Distribution of cepacian biosynthesis genes among environmental and clinical *Burkholderia* strains and role of cepacian exopolysaccharide in resistance to stress conditions. Appl. Environ. Microbiol..

[45] Conway B.-A.D., Chu K.K., Bylund J., Altman E., Speert D.P. (2004). Production of exopolysaccharide by *Burkholderia cenocepacia* results in altered cell-surface interactions and altered bacterial clearance in mice. J. Infect. Dis..

[46] Sousa S.A., Ulrich M., Bragonzi A., Burke M., Worlitzsch D., Leitão J.H., Meisner C., Eberl L., Sá-correia I., Döring G. (2007). Virulence of *Burkholderia cepacia* complex strains in gp91phox-/-mice. Cell. Microbiol..

[47] Bylund J., Burgess L.-A., Cescutti P., Ernst R.K., Speert D.P. (2006). Exopolysaccharides from *Burkholderia cenocepacia* inhibit neutrophil chemotaxis and scavenge reactive oxygen species. J. Biol. Chem..

[48] Cunha M.V., Sousa S.A., Leitão J.H., Moreira L.M., Videira P.A., Sá-Correia I. (2004). Studies on the involvement of the exopolysaccharide produced by cystic fibrosis-associated isolates of the *Burkholderia cepacia* complex in biofilm formation and in persistence of respiratory infections. J. Clin. Microbiol..

[49] Saldías M.S., Lamothe J., Wu R., Valvano M.A. (2008). *Burkholderia cenocepacia* requires the RpoN sigma factor for biofilm formation and intracellular trafficking within macrophages. Infect. Immun..

[50] Hunt T.A., Kooi C., Sokol P.A., Valvano M.A. (2004). Identification of *Burkholderia cenocepacia* genes required for bacterial survival in vivo. Infect. Immun..

[51] Eberl L. (2006). Quorum sensing in the genus *Burkholderia*. Int. J. Med. Microbiol..

[52] Lewenza S., Conway B., Greenberg E.P., Sokol P.A. (1999). Quorum sensing in *Burkholderia cepacia*: Identification of the LuxRI homologs CepRI. J. Bacteriol..

[53] Gotschlich A., Huber B., Geisenberger O., Tögl A., Steidle A., Riedel K., Hill P., Tümmler B., Vandamme P., Middleton B. (2001). Synthesis of Multiple N-Acylhomoserine Lactones is Wide-spread Among the Members of the *Burkholderia cepacia* Complex. Syst. Appl. Microbiol..

[54] Lutter E., Lewenza S., Dennis J.J., Visser M.B., Sokol P.A. (2001). Distribution of Quorum-Sensing Genes in the *Burkholderia cepacia* Complex. Infect. Immun..

[55] Uehlinger S., Schwager S., Bernier S.P., Riedel K., Nguyen D.T., Sokol P.A., Eberl L. (2009). Identification of specific and universal virulence factors in *Burkholderia cenocepacia* strains by using multiple infection hosts. Infect. Immun..

[56] Sokol P.A., Sajjan U., Visser M.B., Gingues S., Forstner J., Kooi C. (2003). The CepIR quorum-sensing system contributes to the virulence of *Burkholderia cenocepacia* respiratory infections. Microbiology.

[57] Subramoni S., Sokol P.A. (2012). Quorum sensing systems influence *Burkholderia cenocepacia* virulence. Future Microbiol..

[58] Venturi V., Friscina A., Bertani I., Devescovi G., Aguilar C. (2004). Quorum sensing in the *Burkholderia cepacia* complex. Res. Microbiol..

[59] Suppiger A., Schmid N., Aguilar C., Pessi G., Eberl L. (2013). Two quorum sensing systems control biofilm formation and virulence in members of the *Burkholderia cepacia* complex. Virulence.

[60] Conway B.-A., Greenberg E.P. (2002). Quorum-sensing signals and quorum-sensing genes in *Burkholderia vietnamiensis*. J. Bacteriol..

[61] Boon C., Deng Y., Wang L.-H., He Y., Xu J.-L., Fan Y., Pan S.Q., Zhang L.-H. (2008). A novel DSF-like signal from *Burkholderia cenocepacia* interferes with *Candida albicans* morphological transition. ISME J..

[62] Deng Y., Wu J., Eberl L., Zhang L.-H. (2010). Structural and Functional Characterization of Diffusible Signal Factor Family Quorum-Sensing Signals Produced by Members of the *Burkholderia cepacia* Complex. Appl. Environ. Microbiol..

[63] Deng Y., Schmid N., Wang C., Wang J., Pessi G., Wu D., Lee J., Aguilar C., Ahrens C.H., Chang C. (2012). Cis-2-dodecenoic acid receptor RpfR links quorum-sensing signal perception with regulation of virulence through cyclic dimeric guanosine monophosphate turnover. Proc. Natl. Acad. Sci. USA.

[64] Ryan R.P., Dow J.M. (2011). Communication with a growing family: Diffusible signal factor (DSF) signaling in bacteria. Trends Microbiol..

[65] Twomey K.B., O’Connell O.J., McCarthy Y., Dow J.M., O’Toole G.A., Plant B.J., Ryan R.P. (2012). Bacterial cis-2-unsaturated fatty acids found in the cystic fibrosis airway modulate virulence and persistence of *Pseudomonas aeruginosa*. ISME J..

[66] Malott R.J., Baldwin A., Mahenthiralingam E., Sokol P.A. (2005). Characterization of the cciIR Quorum-Sensing System in *Burkholderia cenocepacia*. Infect. Immun..

[67] Ryan G.T., Wei Y., Winans S.C. (2013). A LuxR-type repressor of *Burkholderia cenocepacia* inhibits transcription via antiactivation and is inactivated by its cognate acylhomoserine lactone. Mol. Microbiol..

[68] Bernier S.P., Nguyen D.T., Sokol P.A. (2008). A LysR-type transcriptional regulator in *Burkholderia cenocepacia* influences colony morphology and virulence. Infect. Immun..

[69] Aubert D.F., O’Grady E.P., Hamad M.A., Sokol P.A., Valvano M.A. (2013). The *Burkholderia cenocepacia* sensor kinase hybrid AtsR is a global regulator modulating quorum-sensing signalling. Environ. Microbiol..

[70] Khodai-Kalaki M., Aubert D.F., Valvano M.A. (2013). Characterization of the AtsR hybrid sensor kinase phosphorelay pathway and identification of its response regulator in *Burkholderia cenocepacia*. J. Biol. Chem..

[71] Thomas M.S. (2007). Iron acquisition mechanisms of the *Burkholderia cepacia* complex. BioMetals.

[72] Visser M.B., Majumdar S., Hani E., Sokol P.A. (2004). Importance of the ornibactin and pyochelin siderophore transport systems in *Burkholderia cenocepacia* lung infections. Infect. Immun..

[73] Sokol P.A., Darling P., Lewenza S., Corbett C.R., Kooi C.D. (2000). Identification of a siderophore receptor required for ferric ornibactin uptake in *Burkholderia cepacia*. Infect. Immun..

[74] Tyrrell J., Whelan N., Wright C., Sá-Correia I., McClean S., Thomas M., Callaghan M. (2015). Investigation of the multifaceted iron acquisition strategies of *Burkholderia cenocepacia*. BioMetals.

[75] Whitby P.W., VanWagoner T.M., Springer J.M., Morton D.J., Seale T.W., Stull T.L. (2006). *Burkholderia cenocepacia* utilizes ferritin as an iron source. J. Med. Microbiol..

[76] Mathew A., Eberl L., Carlier A.L. (2014). A novel siderophore-independent strategy of iron uptake in the genus *Burkholderia*. Mol. Microbiol..

[77] Bevivino A., Dalmastri C., Tabacchioni S., Chiarini L., Belli M.L., Piana S., Materazzo A., Vandamme P., Manno G. (2002). *Burkholderia cepacia* complex bacteria from clinical and environmental sources in Italy: Genomovar status and distribution of traits related to virulence and transmissibility. J. Clin. Microbiol..

[78] Hutchison M.L., Poxton I.R., Govan J.R. (1998). *Burkholderia cepacia* produces a hemolysin that is capable of inducing apoptosis and degranulation of mammalian phagocytes. Infect. Immun..

[79] Thomson E.L.S., Dennis J.J. (2012). A *Burkholderia cepacia* complex non-ribosomal peptide-synthesized toxin is hemolytic and required for full virulence. Virulence.

[80] Ramos J.L., Martínez-Bueno M., Molina-Henares A.J., Terán W., Watanabe K., Zhang X., Gallegos M.T., Brennan R., Tobes R. (2005). The TetR family of transcriptional repressors. Microbiol. Mol. Biol. Rev..

[81] Winsor G.L., Khaira B., van Rossum T., Lo R., Whiteside M.D., Brinkman F.S.L. (2008). The *Burkholderia* Genome Database: Facilitating flexible queries and comparative analyses. Bioinformatics.

[82] O’Grady E.P., Nguyen D.T., Weisskopf L., Eberl L., Sokol P.A. (2011). The *Burkholderia cenocepacia* LysR-type transcriptional regulator ShvR influences expression of quorum-sensing, protease, type II secretion, and afc genes. J. Bacteriol..

[83] Thomson E.L.S., Dennis J.J. (2013). Common duckweed (*Lemna minor*) is a versatile high-throughput infection model for the *Burkholderia cepacia* complex and other pathogenic bacteria. PLoS ONE.

[84] Subramoni S., Agnoli K., Eberl L., Lewenza S., Sokol P.A. (2013). Role of *Burkholderia cenocepacia afcE* and *afcF* genes in determining lipid-metabolism-associated phenotypes. Microbiology.

[85] Michalska K., Chhor G., Clancy S., Jedrzejczak R., Babnigg G., Winans S.C., Joachimiak A. (2014). RsaM: A transcriptional regulator of *Burkholderia* spp. with novel fold. FEBS J..

[86] Stock A.M., Robinson V.L., Goudreau P.N. (2000). Two-Component Signal Transduction. Annu. Rev. Biochem..

[87] Merry C.R., Perkins M., Mu L., Peterson B.K., Knackstedt R.W., Weingart C.L. (2015). Characterization of a Novel Two-Component System in *Burkholderia cenocepacia*. Curr. Microbiol..

[88] Gislason A.S., Choy M., Bloodworth R.A.M., Qu W., Stietz M.S., Li X., Zhang C., Cardona S.T. (2016). Competitive growth enhances conditional growth mutant sensitivity to antibiotics and exposes a two-component system as an emerging antibacterial target in *Burkholderia cenocepacia*. Antimicrob. Agents Chemother..

[89] Aubert D.F., Valvano M.A., Hu S. (2015). Quantification of type VI secretion system activity in macrophages infected with *Burkholderia cenocepacia*. Microbiology.

[90] Flannagan R.S., Aubert D., Kooi C., Sokol P.A., Valvano M.A. (2007). *Burkholderia cenocepacia* requires a periplasmic HtrA protease for growth under thermal and osmotic stress and for survival in vivo. Infect. Immun..

[91] Schaefers M.M., Liao T.L., Boisvert N.M., Roux D., Yoder-Himes D., Priebe G.P. (2017). An Oxygen-Sensing Two-Component System in the *Burkholderia cepacia* Complex Regulates Biofilm, Intracellular Invasion, and Pathogenicity. PLoS Pathog..

[92] Wen J., Fozo E. (2014). sRNA Antitoxins: More than One Way to Repress a Toxin. Toxins.

[93] Lobato-Márquez D., Díaz-Orejas R., García-del Portillo F. (2016). Toxin-antitoxins and bacterial virulence. FEMS Microbiol. Rev..

[94] Gottesman S. (2005). Micros for microbes: Non-coding regulatory RNAs in bacteria. Trends Genet..

[95] Papenfort K., Vogel J. (2010). Regulatory RNA in Bacterial Pathogens. Cell Host Microbe.

[96] Feliciano J.R., Grilo A.M., Guerreiro S.I., Sousa S.A., Leitão J.H. (2016). Hfq: A multifaceted RNA chaperone involved in virulence. Future Microbiol..

[97] Updegrove T.B., Zhang A., Storz G. (2016). Hfq: The flexible RNA matchmaker. Curr. Opin. Microbiol..

[98] Sousa S.A., Ramos C.G., Moreira L.M., Leitão J.H. (2010). The hfq gene is required for stress resistance and full virulence of *Burkholderia cepacia* to the nematode *Caenorhabditis elegans*. Microbiology.

[99] Ramos C.G., Grilo A.M., da Costa P.J.P., Leitão J.H. (2013). Experimental identification of small non-coding regulatory RNAs in the opportunistic human pathogen *Burkholderia cenocepacia* J2315. Genomics.

[100] Ramos C.G., da Costa P.J.P., Döring G., Leitão J.H. (2012). The Novel Cis-Encoded Small RNA h2cR Is a Negative Regulator of hfq2 in *Burkholderia cenocepacia*. PLoS ONE.

[101] Ramos C.G., Grilo A.M., Sousa S.A., Feliciano J.R., Da Costa P.J.P., Leitão J.H. (2014). Regulation of Hfq mRNA and protein levels in *Escherichia coli* and *Pseudomonas aeruginosa* by the *Burkholderia cenocepacia* MtvR sRNA. PLoS ONE.

[102] Kimelman A., Levy A., Sberro H., Kidron S., Leavitt A., Amitai G., Yoder-Himes D.R., Wurtzel O., Zhu Y., Rubin E.M. (2012). A vast collection of microbial genes that are toxic to bacteria. Genome Res..

